# Supplementary material for: Distinct cellular immune responses in children en route to type 1 diabetes with different first-appearing autoantibodies
Source: Nat Commun. 2024 May 7;15:3810. doi: 10.1038/s41467-024-47918-w (PMC11076468; doi:10.1038/s41467-024-47918-w)
Supplement: Supplementary file 1 — Supplementary Information [file 41467_2024_47918_MOESM1_ESM.pdf]

Distinct cellular immune responses in children en route to type 1 diabetes with different first-appearing autoantibodies

Inna Starskaia[1,2,3], Milla Valta[3,4], Sami Pietilä[1,2], Tomi Suomi[1,2], Sirpa Pahkuri[3,4], Ubaid Ullah Kalim[1,2], Omid Rasool[1,2], Emilie Rydgren[1,2], Heikki Hyöty[5], Mikael Knip[6,7], Riitta Veijola[8], Jorma Ilonen[4], Jorma Toppari[2,9,10,11], Johanna Lempainen[4,11,12*], Laura L. Elo[1,2,13*], and Riitta Lahesmaa[1,2,13*]

[1]Turku Bioscience Centre, University of Turku and Åbo Akademi University, Turku, Finland
[2]InFLAMES Research Flagship Center, University of Turku
[3]Turku Doctoral Programme of Molecular Medicine, University of Turku, Turku, Finland
[4]Immunogenetics Laboratory, Institute of Biomedicine, University of Turku, Turku, Finland
[5]Faculty of Medicine and Health Technology, Tampere University, and Fimlab Laboratories, Tampere, Finland
[6]Research Program for Clinical and Molecular Metabolism, Faculty of Medicine, University of Helsinki, Helsinki, Finland
[7]Center for Child Health Research, Tampere University Hospital, Tampere, Finland
[8]Department of Pediatrics, Research Unit of Clinical Medicine, Medical Research Centre, Oulu University Hospital and University of Oulu, Oulu, Finland
[9]Centre for Population Health Research, University of Turku and Turku University Hospital, Turku, Finland
[10]Research Centre for Integrative Physiology and Pharmacology, Institute of Biomedicine, University of Turku, Turku, Finland
[11]Department of Pediatrics, University of Turku and Turku University Hospital, Turku, Finland
[12]Clinical Microbiology, Turku University Hospital, Turku, Finland
[13]Institute of Biomedicine, University of Turku, Turku, Finland

These authors contributed equally: Inna Starskaia, Milla Valta, Sami Pietilä, Tomi Suomi
Corresponding authors: johanna.lempainen@utu.fi, laura.elo@utu.fi, rilahes@utu.fi

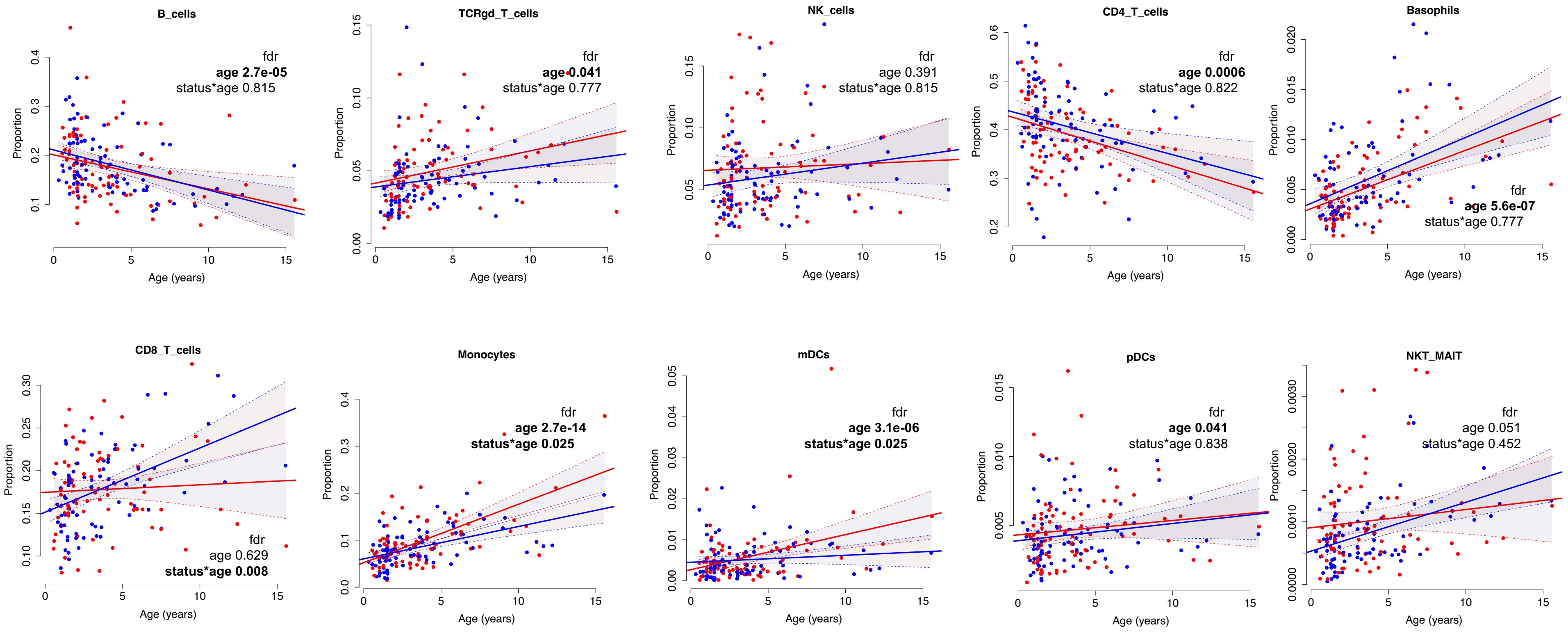

Supplementary Figure 1. **Cell type proportions of main PBMC subsets from the children of all three groups**. Scatter plots of the main PBMC subsets over time. Each plot is annotated with the mixed effects model FDR for age and its interaction with status (status*age). Red and blue dots indicate cases and controls samples, respectively. Red and blue lines show linear regression fit with 95% confident intervals for cases and controls, respectively. Statistical analyses were performed using linear mixed effects modeling. Multiple test corrections have been applied for p values using the Benjamini–Hochberg method.

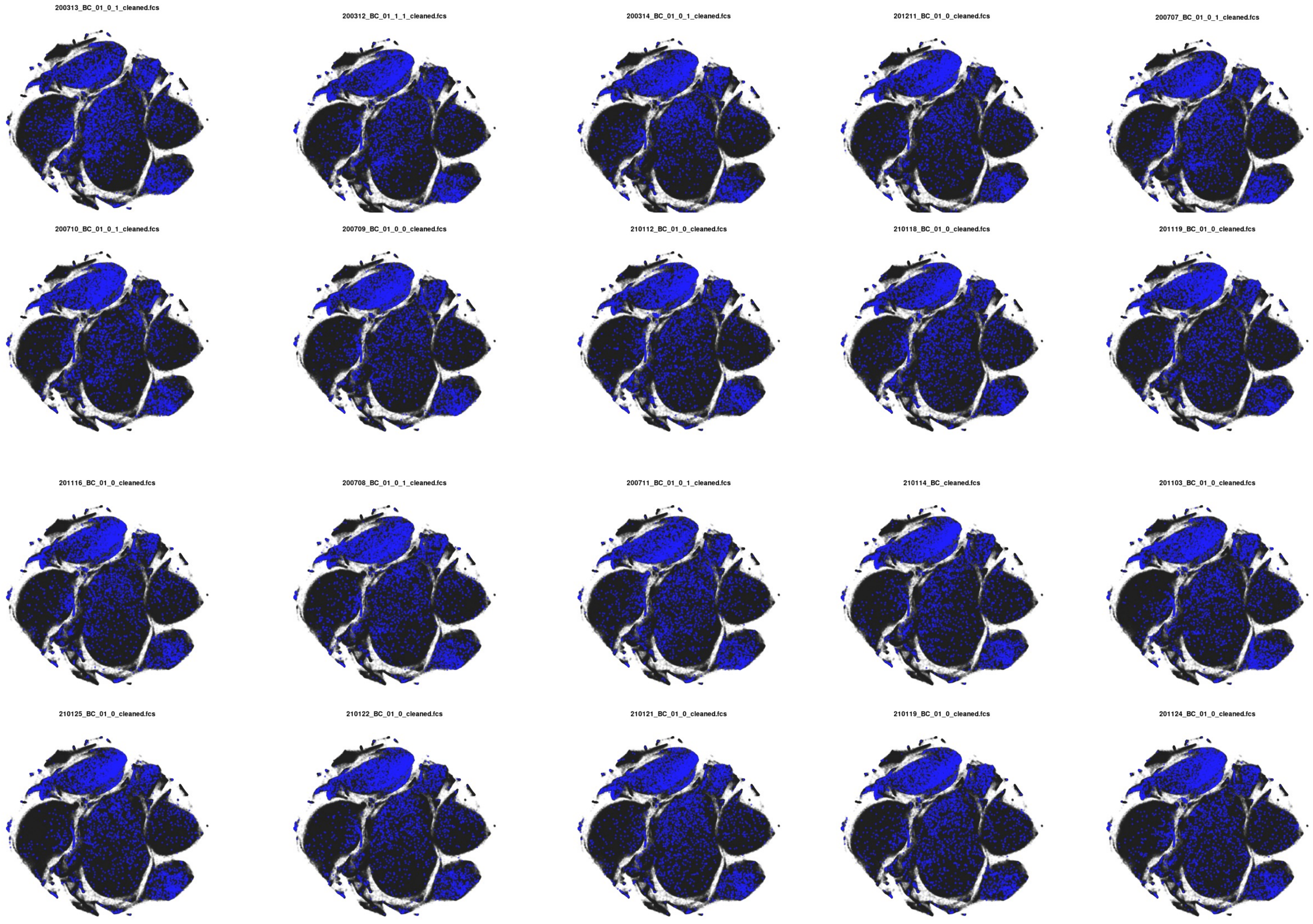

Supplementary Figure 2. t-SNE plot of batch control samples.

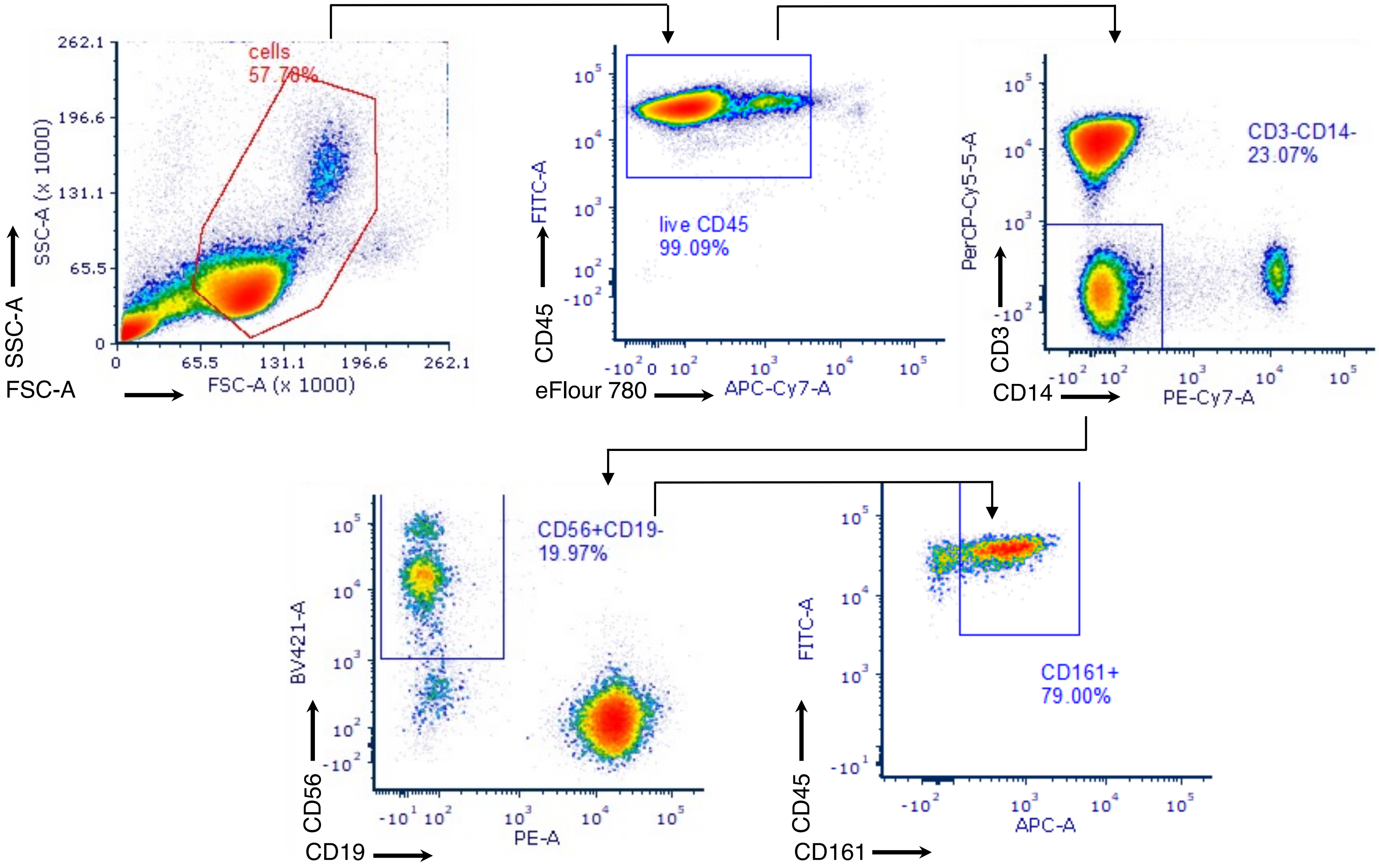

Supplementary Figure 3. Gating strategy of CD161 expression in NK cells from PBMC samples of children from the validation cohort by flow cytometry in Figure 6a, b.

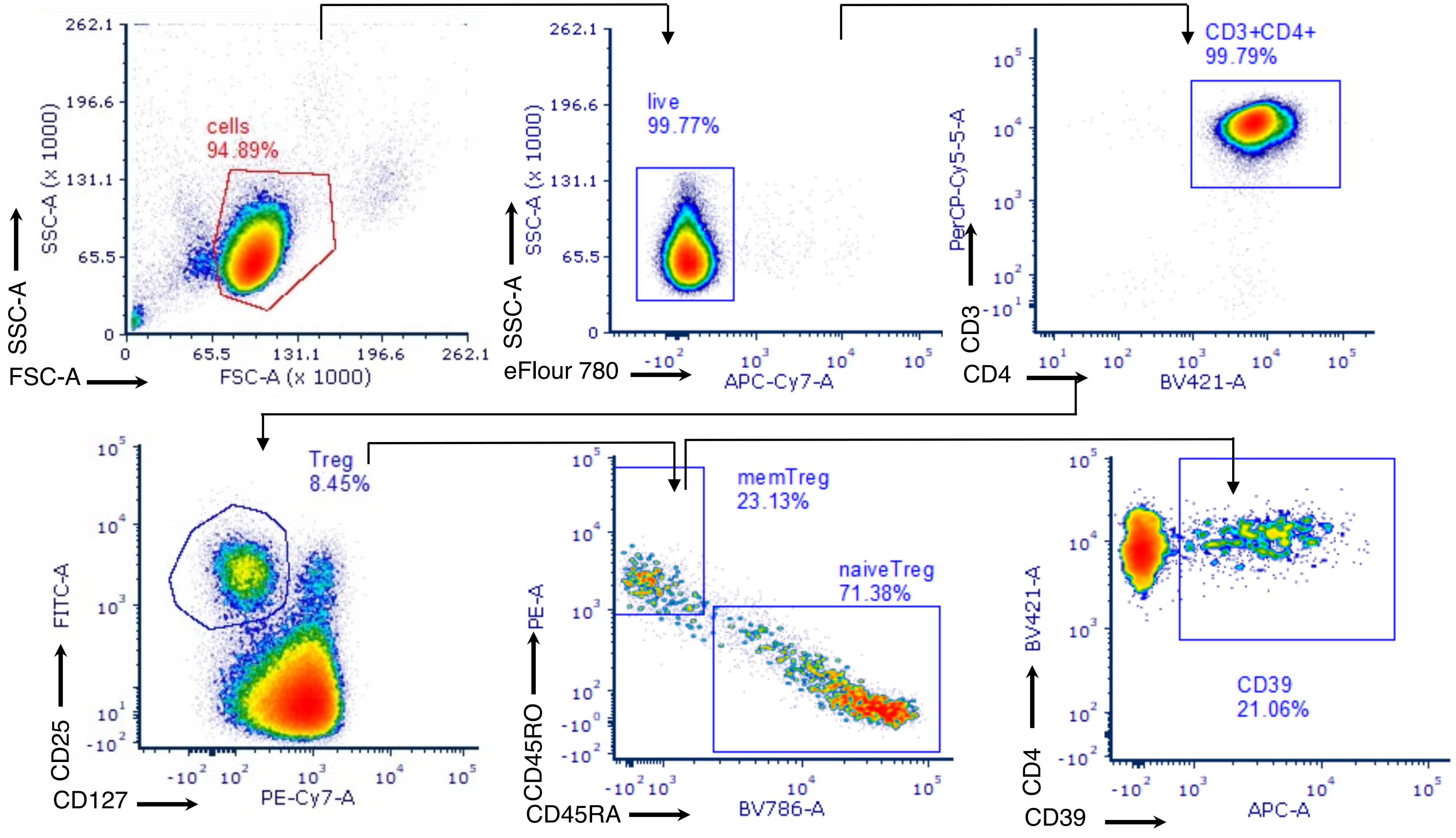

Supplementary Figure 4. Gating strategy of CD39 expression in memory Treg from CD4+ T cell fraction of children from the validation cohort by flow cytometry in Figure 6c, d.

Supplementary Table 1. The list of antibodies.

Maxpar Direct Immune Profiling assay (Standard BioTools, cat # 201325):

Target	Metal	Clone
CD45	89Y	HI30
CD196/CCR6	141Pr	G034E3
CD123	143Nd	6H6
CD19	144Nd	HIB19
CD4	145Nd	RPA-T4
CD8a	146Nd	RPA-T8
CD11c	147Sm	Bu15
CD16	148Nd	3G8
CD45RO	149Sm	UCHL1
CD45RA	150Nd	HI100
CD161	151Eu	HP-3G10
CD194/CCR4	152Sm	L291H4
CD25	153Eu	BC96
CD27	154Sm	O323
CD57	155Gd	HCD57
CD183/CXCR3	156Gd	G025H7
CD185/CXCR5	158Gd	J252D4
CD28	160Gd	CD28.2
CD38	161Dy	HB-7
CD56/NCAM	163Dy	NCAM16.2
TCRgd	164Dy	B1
CD294	166Er	BM16
CD197/CCR7	167Er	G043H7
CD14	168Er	63D3
CD3	170Er	UCHT1

CD20	171Yb	2H7
CD66b	172Yb	G10F5
HLA-DR	173Yb	LN3
IgD	174Yb	IA6-2
CD127	176Yb	A019D5
Live/dead intercalator	103Rh	

Additional antibodies, ready-made

Target	Concentration	Metal	Clone	cat #
LAG-3	1:100	165Ho	11C3C65	St. BioTools, cat # 3165037B
CD69	1:200	162Dy	FN50	St. BioTools, cat # 3162001B
PD-1	1:200	175Lu	EH12.2H7	St. BioTools, cat # 3175008B

Additional antibodies, in-house conjugated

Target	Concentration	Metal	Clone	cat #
ICOS	1:200	111Cd	C398.4A	BioLegend, cat # 313502
Cd15s	1:200	113Cd	CSLEX1	BD Biosciences, cat # 551344
CD39	1:200	116Cd	A1	BioLegend, cat #328221
CTLA-4	1:100	142Nd	14D3	Invitrogen, cat #14-1529-82
TIGIT	1:200	159Tb	VSTM3	BioLegend, cat # 372702
CCR10	1:100	169Tm	6588-5	BioLegend, cat # 341502

Supplementary Table 2. LME results in cell type proportions across all samples

	**Coefficent**						**P value**						**FDR**					
Cell type	age	sex	HLA-DR3/4	HLA-DR4	**status**	**age*status**	age	sex	HLA-DR3/4	HLA-DR4	**status**	**age*status**	age	sex	HLA-DR3/4	HLA-DR4	**status**	**age*status**
B cells	-0,0090	-0,0005	-0,0132	-0,0313	0,0089	-0,0012	1,11E-05	0,9757	0,3769	0,0279	0,4670	0,6520	2,77E-05	0,9757	0,6795	0,0930	0,6583	0,8151
CD8+ T cells	0,0007	0,0206	-0,0064	0,0173	-0,0247	0,0070	0,6293	0,0597	0,5436	0,0908	0,0119	0,0008	0,6293	0,4235	0,6795	0,1817	0,0856	0,0079
TCRgd T cells	0,0017	0,0038	0,0040	0,0070	-0,0024	-0,0007	0,0291	0,4881	0,4475	0,1718	0,6111	0,4662	0,0415	0,8459	0,6795	0,2455	0,6790	0,7771
NKT&MAIT	0,0000	0,0000	0,0001	0,0001	-0,0003	0,0000	0,0410	0,9299	0,5111	0,6574	0,0171	0,1808	0,0512	0,9757	0,6795	0,7305	0,0856	0,4521
NK cells	0,0011	0,0015	-0,0029	-0,0001	-0,0107	0,0009	0,3520	0,8533	0,7234	0,9880	0,1522	0,5820	0,3911	0,9757	0,8038	0,9880	0,3805	0,8151
pDCs	0,0002	-0,0005	-0,0013	-0,0008	-0,0003	0,0000	0,0256	0,5076	0,0346	0,1485	0,5266	0,8383	0,0415	0,8459	0,3457	0,2455	0,6583	0,8383
CD4+ T cells	-0,0091	-0,0270	0,0139	-0,0170	0,0118	0,0011	0,0003	0,1694	0,4404	0,3205	0,4450	0,7399	0,0006	0,4235	0,6795	0,4006	0,6583	0,8221
Basophils	0,0006	0,0014	0,0007	0,0015	0,0002	0,0001	1,12E-07	0,1558	0,3698	0,0563	0,7836	0,4474	5,60E-07	0,4235	0,6795	0,1407	0,7836	0,7771
Monocytes	0,0127	0,0017	0,0105	0,0209	0,0085	-0,0055	2,70E-15	0,8409	0,2491	0,0223	0,3530	0,0057	2,70E-14	0,9757	0,6795	0,0930	0,6583	0,0257
mDCs	0,0010	-0,0020	0,0002	0,0029	0,0020	-0,0007	9,24E-07	0,0944	0,8710	0,0171	0,1072	0,0077	3,08E-06	0,4235	0,8710	0,0930	0,3573	0,0257

Supplementary Table 3. LME results in cell type proportions in GADA-firts, IAA-first, and ≥2 Aab first groups

	Coefficent									
Cell type	age	sex	**GADA**	**IAA**	**≥ 2 Aab**	HLA-DR3/4	HLA-DR4	**age*GADA**	**age*IAA**	**age*≥2 Aab**
B cells	-0,0092	0,0038	-0,0408	0,0197	0,0261	-0,0095	-0,0194	0,0005	-0,0070	0,0023
CD8+ T cells	0,0074	0,0179	0,0431	-0,0060	0,0129	-0,0069	0,0158	-0,0077	0,0026	-0,0099
TCRgd T cells	0,0011	0,0066	0,0016	-0,0124	0,0235	0,0086	0,0116	0,0001	0,0014	-0,0004
NKT&MAIT	0,0001	0,0000	0,0006	-0,0001	0,0002	0,0001	0,0000	-0,0001	0,0001	-0,0001
NK cells	0,0021	0,0048	0,0245	-0,0223	0,0283	0,0052	0,0069	-0,0022	0,0020	-0,0023
pDCs	0,0002	-0,0002	0,0001	-0,0006	0,0010	-0,0009	-0,0006	0,0002	0,0001	-0,0001
CD4+ T cells	-0,0083	-0,0421	-0,0089	0,0371	-0,0744	-0,0033	-0,0326	-0,0019	-0,0019	0,0026
Basophils	0,0007	0,0014	0,0001	-0,0012	0,0004	0,0007	0,0015	-0,0003	0,0002	-0,0002
Monocytes	0,0071	0,0037	-0,0008	-0,0161	-0,0086	0,0147	0,0221	0,0071	0,0027	0,0062
mDCs	0,0002	-0,0014	-0,0057	-0,0005	-0,0020	0,0006	0,0026	0,0025	0,0001	0,0004
	**P value**									
B cells	0,0000	0,8145	0,0212	0,2960	0,1507	0,5213	0,1610	0,9126	0,0791	0,4562
CD8+ T cells	0,0000	0,0896	0,0015	0,6740	0,3435	0,5011	0,1072	0,0183	0,3888	0,0001
TCRgd T cells	0,1312	0,2127	0,8088	0,0858	0,0007	0,0990	0,0200	0,9416	0,3773	0,7355
NKT&MAIT	0,0003	0,9048	0,0022	0,6264	0,2441	0,5949	0,9417	0,1996	0,1261	0,0646
NK cells	0,0679	0,5692	0,0230	0,0524	0,0120	0,5375	0,3846	0,3879	0,4110	0,2309
pDCs	0,0546	0,7682	0,8915	0,4508	0,1793	0,1473	0,2930	0,1842	0,6097	0,3886
CD4+ T cells	0,0004	0,0413	0,6828	0,1106	0,0011	0,8580	0,0557	0,7212	0,7037	0,4950
Basophils	0,0000	0,1671	0,8975	0,2837	0,7153	0,4264	0,0617	0,2626	0,4331	0,2722
Monocytes	0,0000	0,6612	0,9535	0,2567	0,5235	0,1132	0,0149	0,0305	0,3792	0,0109
mDCs	0,1733	0,2334	0,0007	0,7651	0,2352	0,5979	0,0264	0,0000	0,8801	0,2271
	**FDR**									
B cells	5,1E-06	0,9048	0,0460	0,4934	0,3487	0,6644	0,2300	0,9416	0,6188	0,5500
CD8+ T cells	2,3E-06	0,4481	0,0072	0,7488	0,4293	0,6644	0,1787	0,0917	0,6188	0,0005
TCRgd T cells	0,1457	0,4667	0,9535	0,3687	0,0053	0,4912	0,0880	0,9416	0,6188	0,7355
NKT&MAIT	0,0007	0,9048	0,0072	0,7488	0,3487	0,6644	0,9417	0,3992	0,6188	0,2153
NK cells	0,0849	0,9048	0,0460	0,3687	0,0399	0,6644	0,4274	0,5542	0,6188	0,4537
pDCs	0,0780	0,9048	0,9535	0,6440	0,3487	0,4912	0,3662	0,3992	0,7622	0,5500
CD4+ T cells	0,0007	0,4129	0,9535	0,3687	0,0053	0,8580	0,1235	0,9015	0,7819	0,5500
Basophils	4,7E-09	0,4667	0,9535	0,4934	0,7153	0,6644	0,1235	0,4377	0,6188	0,4537
Monocytes	2,9E-06	0,9048	0,9535	0,4934	0,5816	0,4912	0,0880	0,1018	0,6188	0,0545
mDCs	0,17329	0,4667	0,0068	0,7651	0,3487	0,6644	0,0880	3,0E-08	0,8801	0,4537

Supplementary Table 4. Models comparison results

cell type	better model	p
B cells	2	**0,000**
CD8+ T cells	2	**0,000**
TCRgd T cells	2	**0,000**
NKT&MAIT	2	**0,022**
NK cells	2	**0,008**
pDCs	0	0,203
CD4+ T cells	2	**0,001**
Basophils	0	0,558
Monocytes	0	0,150
mDCs	2	**0,000**

0 No significant difference
1 All groups model is better
2 Subset groups model is better
