# Supplementary material for: Distinct cellular immune responses in children en route to type 1 diabetes with different first-appearing autoantibodies
Source: Nat Commun. 2024 May 7;15:3810. doi: 10.1038/s41467-024-47918-w (PMC11076468; doi:10.1038/s41467-024-47918-w)
Supplement: Supplementary file 3 — Description of additional supplementary files [file 41467_2024_47918_MOESM3_ESM.docx]

**Description of additional supplementary files**

**Supplementary Data 1. LME results of marker expression in main PBMC subsets.** The file contains the LME results of marker expression in the main PBMC subsets in ≥2 Aab, IAA-, GADA-first, and all samples analyses groups. The data for each group presented on a separate sheet. LME, linear mixed effects modelling, PBMC, peripheral blood mononuclear cells, FDR, false discovery rate.

**Supplementary Data 1. LME results of marker expression in CD4+ and CD8+ T cell subsets.** The file contains the LME results of marker expression in CD4+ and CD8+ T cell subsets in ≥2 Aab, IAA-, GADA-first, and all samples analyses groups. The data for each cell subset and group presented on a separate sheet. LME, linear mixed effects modelling, PBMC, peripheral blood mononuclear cells, FDR, false discovery rate.
