# Supplementary material for: Distinct cellular immune responses in children en route to type 1 diabetes with different first-appearing autoantibodies
Source: Nat Commun. 2024 May 7;15:3810. doi: 10.1038/s41467-024-47918-w (PMC11076468; doi:10.1038/s41467-024-47918-w)
Supplement: Supplementary file 6 — Reporting Summary [file 41467_2024_47918_MOESM6_ESM.pdf]

nature portfolio

Corresponding author(s): Riitta Lahesmaa

Last updated by author(s): Mar 22, 2024

Reporting Summary

Nature Portfolio wishes to improve the reproducibility of the work that we publish. This form provides structure for consistency and transparency in reporting. For further information on Nature Portfolio policies, see our Editorial Policies and the Editorial Policy Checklist.

Statistics

For all statistical analyses, confirm that the following items are present in the figure legend, table legend, main text, or Methods section.

n/a	Confirmed	
☐	☒	The exact sample size (*n*) for each experimental group/condition, given as a discrete number and unit of measurement
☐	☒	A statement on whether measurements were taken from distinct samples or whether the same sample was measured repeatedly
☐	☒	The statistical test(s) used AND whether they are one- or two-sided<br>*Only common tests should be described solely by name; describe more complex techniques in the Methods section.*
☐	☒	A description of all covariates tested
☐	☒	A description of any assumptions or corrections, such as tests of normality and adjustment for multiple comparisons
☐	☒	A full description of the statistical parameters including central tendency (e.g. means) or other basic estimates (e.g. regression coefficient) AND variation (e.g. standard deviation) or associated estimates of uncertainty (e.g. confidence intervals)
☐	☒	For null hypothesis testing, the test statistic (e.g. *F*, *t*, *r*) with confidence intervals, effect sizes, degrees of freedom and *P* value noted<br>*Give P values as exact values whenever suitable.*
☒	☐	For Bayesian analysis, information on the choice of priors and Markov chain Monte Carlo settings
☒	☐	For hierarchical and complex designs, identification of the appropriate level for tests and full reporting of outcomes
☒	☐	Estimates of effect sizes (e.g. Cohen's *d*, Pearson's *r*), indicating how they were calculated

Our web collection on statistics for biologists contains articles on many of the points above.

Software and code

Policy information about availability of computer code

Data collection	CyTOF Software v. 6 was used acquisition of mass cytometry experiments, BD FACSDiva™ Software v. 9 was used for acquisition of flow cytometry experiments.
Data analysis	For mass cytometry data pre-processing and cleanup: Maxpar Pathsetter version 2.0; for mass cytometry data analsysis: R language version 4.3.0.; flowCore version 2.12.2; flowSOM version 2.8.0; ConsensusClusterPlus version 1.64.0; lmerTest version 3.1-3. For flow cytometry data analysis: FCS Express 7 Flow version 7.16.0046.<br>The R scripts generated during this study are available at Github repository (https://github.com/elolab/T1D-CyTOF-analysis).

For manuscripts utilizing custom algorithms or software that are central to the research but not yet described in published literature, software must be made available to editors and reviewers. We strongly encourage code deposition in a community repository (e.g. GitHub). See the Nature Portfolio guidelines for submitting code & software for further information.

Data

Policy information about availability of data

All manuscripts must include a data availability statement. This statement should provide the following information, where applicable:

- Accession codes, unique identifiers, or web links for publicly available datasets
- A description of any restrictions on data availability
- For clinical datasets or third party data, please ensure that the statement adheres to our policy

The raw epidemiological and generated data are protected and are not available due to data privacy laws. The data processed in this study and underlying figures and tables are provided in the Supplementary Information/Source Data file. Source data are provided with this paper.

Research involving human participants, their data, or biological material

Policy information about studies with human participants or human data. See also policy information about sex, gender (identity/presentation), and sexual orientation and race, ethnicity and racism.

Reporting on sex and gender	Sex information was collected and considered in study design, the case-control pairs were matched by sex. Further, in linear mixed effect model, sex was treated as fixed effects. Sex was assigned at birth by the midwife based on the appearance of the child.
Reporting on race, ethnicity, or other socially relevant groupings	NA
Population characteristics	The detailed characteristics of study participants can be found in the Methods sections and Table 1.
Recruitment	All study subjects were participants in the Finnish Type 1 Diabetes Prediction and Prevention (DIPP) study. This ongoing, prospective follow-up study recruits newborns based on HLA-conferred genetic susceptility to type 1 diabetes at Turku, Oulu and Tampere University Hospitals. Further information on the study cohort can be found in the Material and methods section.
Ethics oversight	The study was originally approved by the Ethics Committee of the Hospital District of Southwest Finland, followed by the Ethics Committee of the Hospital District of Northern Ostrobothnia, and written informed consent was provided by the families participating in the study.

Note that full information on the approval of the study protocol must also be provided in the manuscript.

Field-specific reporting

Please select the one below that is the best fit for your research. If you are not sure, read the appropriate sections before making your selection.

☒ Life sciences ☐ Behavioural & social sciences ☐ Ecological, evolutionary & environmental sciences

For a reference copy of the document with all sections, see nature.com/documents/nr-reporting-summary-flat.pdf

Life sciences study design

All studies must disclose on these points even when the disclosure is negative.

Sample size	We acquired samples from 29 children who later progressed to the clinical type 1 diabetes and their carefully matched autoantibody-negative control children by age, HLA, and sex in the discovery cohort (11, 9, and 9 case-control pairs for IAA-first, GADA-first, ≥2 autoantibodies first). From each child we had 3-4 follow-up samples. No such previous studies were carried out previously and prior statistical tests to predetermine sample size was not possible. To achieve our samples series, PBMCs from the children participating in the DIPP study have been collected during a period of 20 years. We had at least 9 case-control pairs available for each autoantibody group and several time points were analyzed for each individual to ensure sufficient sample size.
Data exclusions	10 samples were excluded from the data analysis due to technical issues occurred during data acquisition of these samples on the mass cytometer.
Replication	An independent validation cohort consisting of 30 children (10 cases in each autoantibody subgroup) who had developed beta-cell autoimmunity or progressed to clinical type 1 diabetes and 30 matched controls. Due to sample material and technology limitations, we selected only two findings for validation based on their expression level and statistical significance: CD161 in NK cells and CD39 in Treg cells. The results were validated for one of the findings, confirming increased expression of CD161 in NK cells was in children positive for ≥2 Aab than in controls. However, we were not able to validate the results of CD39 expression in Treg cells.
Randomization	The samples have been randomized within each experiment group. All samples were divided in three groups based on autoantibody type (IAA, GADA, ≥2 Aab groups). Both the technical batch and the Aab group were included to the model to account for potential batch effect.

	Moreover, samples from each case–control pair were processed at the same time (including thawing, staining, and acquisition), and it was accounted by having the pair as a random effect in the mixed effects model.
Blinding	The experiment was carried out without blinding. Samples were acquired from 29 children participating in the DIPP study who later progressed to the clinical type 1 diabetes, together with their carefully matched autoantibody-negative controls.

Behavioural & social sciences study design

All studies must disclose on these points even when the disclosure is negative.

Study description	*Briefly describe the study type including whether data are quantitative, qualitative, or mixed-methods (e.g. qualitative cross-sectional, quantitative experimental, mixed-methods case study).*
Research sample	*State the research sample (e.g. Harvard university undergraduates, villagers in rural India) and provide relevant demographic information (e.g. age, sex) and indicate whether the sample is representative. Provide a rationale for the study sample chosen. For studies involving existing datasets, please describe the dataset and source.*
Sampling strategy	*Describe the sampling procedure (e.g. random, snowball, stratified, convenience). Describe the statistical methods that were used to predetermine sample size OR if no sample-size calculation was performed, describe how sample sizes were chosen and provide a rationale for why these sample sizes are sufficient. For qualitative data, please indicate whether data saturation was considered, and what criteria were used to decide that no further sampling was needed.*
Data collection	*Provide details about the data collection procedure, including the instruments or devices used to record the data (e.g. pen and paper, computer, eye tracker, video or audio equipment) whether anyone was present besides the participant(s) and the researcher, and whether the researcher was blind to experimental condition and/or the study hypothesis during data collection.*
Timing	*Indicate the start and stop dates of data collection. If there is a gap between collection periods, state the dates for each sample cohort.*
Data exclusions	*If no data were excluded from the analyses, state so OR if data were excluded, provide the exact number of exclusions and the rationale behind them, indicating whether exclusion criteria were pre-established.*
Non-participation	*State how many participants dropped out/declined participation and the reason(s) given OR provide response rate OR state that no participants dropped out/declined participation.*
Randomization	*If participants were not allocated into experimental groups, state so OR describe how participants were allocated to groups, and if allocation was not random, describe how covariates were controlled.*

Ecological, evolutionary & environmental sciences study design

All studies must disclose on these points even when the disclosure is negative.

Study description	*Briefly describe the study. For quantitative data include treatment factors and interactions, design structure (e.g. factorial, nested, hierarchical), nature and number of experimental units and replicates.*
Research sample	*Describe the research sample (e.g. a group of tagged Passer domesticus, all Stenocereus thurberi within Organ Pipe Cactus National Monument), and provide a rationale for the sample choice. When relevant, describe the organism taxa, source, sex, age range and any manipulations. State what population the sample is meant to represent when applicable. For studies involving existing datasets, describe the data and its source.*
Sampling strategy	*Note the sampling procedure. Describe the statistical methods that were used to predetermine sample size OR if no sample-size calculation was performed, describe how sample sizes were chosen and provide a rationale for why these sample sizes are sufficient.*
Data collection	*Describe the data collection procedure, including who recorded the data and how.*
Timing and spatial scale	*Indicate the start and stop dates of data collection, noting the frequency and periodicity of sampling and providing a rationale for these choices. If there is a gap between collection periods, state the dates for each sample cohort. Specify the spatial scale from which the data are taken*
Data exclusions	*If no data were excluded from the analyses, state so OR if data were excluded, describe the exclusions and the rationale behind them, indicating whether exclusion criteria were pre-established.*
Reproducibility	*Describe the measures taken to verify the reproducibility of experimental findings. For each experiment, note whether any attempts to repeat the experiment failed OR state that all attempts to repeat the experiment were successful.*
Randomization	*Describe how samples/organisms/participants were allocated into groups. If allocation was not random, describe how covariates were controlled. If this is not relevant to your study, explain why.*
Blinding	*Describe the extent of blinding used during data acquisition and analysis. If blinding was not possible, describe why OR explain why blinding was not relevant to your study.*

Did the study involve field work? ☐ Yes ☐ No

Field work, collection and transport

Field conditions	*Describe the study conditions for field work, providing relevant parameters (e.g. temperature, rainfall).*
Location	*State the location of the sampling or experiment, providing relevant parameters (e.g. latitude and longitude, elevation, water depth).*
Access & import/export	*Describe the efforts you have made to access habitats and to collect and import/export your samples in a responsible manner and in compliance with local, national and international laws, noting any permits that were obtained (give the name of the issuing authority, the date of issue, and any identifying information).*
Disturbance	*Describe any disturbance caused by the study and how it was minimized.*

Reporting for specific materials, systems and methods

We require information from authors about some types of materials, experimental systems and methods used in many studies. Here, indicate whether each material, system or method listed is relevant to your study. If you are not sure if a list item applies to your research, read the appropriate section before selecting a response.

Materials & experimental systems

n/a	Involved in the study	
☐	☒	Antibodies
☒	☐	Eukaryotic cell lines
☒	☐	Palaeontology and archaeology
☒	☐	Animals and other organisms
☒	☐	Clinical data
☒	☐	Dual use research of concern
☒	☐	Plants

Methods

n/a	Involved in the study	
☒	☐	ChIP-seq
☐	☒	Flow cytometry
☒	☐	MRI-based neuroimaging

Antibodies

Antibodies used

The list of antibodies (also can be found in the Supplementary material)
Maxpar Direct Immune Profiling assay (Standard BioTools, cat # 201325):
Target | Metal | Clone
CD45 | 89Y | HI30
CD196/CCR6 | 141Pr | G034E3
CD123 | 143Nd | 6H6
CD19 | 144Nd | HIB19
CD4 | 145Nd | RPA-T4
CD8a | 146Nd | RPA-T8
CD11c | 147Sm | Bu15
CD16 | 148Nd | 3G8
CD45RO | 149Sm | UCHL1
CD45RA | 150Nd | HI100
CD161 | 151Eu | HP-3G10
CD194/CCR4 | 152Sm | L291H4
CD25 | 153Eu | BC96
CD27 | 154Sm | O323
CD57 | 155Gd | HCD57
CD183/CXCR3 | 156Gd | G025H7
CD185/CXCR5 | 158Gd | J252D4
CD28 | 160Gd | CD28.2
CD38 | 161Dy | HB-7
CD56/NCAM | 163Dy | NCAM16.2
TCRgd | 164Dy | B1
CD294 | 166Er | BM16
CD197/CCR7 | 167Er | G043H7
CD14 | 168Er | 63D3
CD3 | 170Er | UCHT1
CD20 | 171Yb | 2H7
CD66b | 172Yb | G10F5
HLA-DR | 173Yb | LN3
IgD | 174Yb | IA6-2

CD127 | 176Yb | A019D5
Live/dead intercalator | 103Rh

Additional antibodies, ready-made
Target | Concentration | Metal | Clone | cat #
LAG-3 | 1:100 | 165Ho | 11C3C65 | St. BioTools, cat # 3165037B
CD69 | 1:200 | 162Dy | FN50 | St. BioTools, cat # 3162001B
PD-1 | 1:200 | 175Lu | EH12.2H7 | St. BioTools, cat # 3175008B

Additional antibodies, in-house conjugated
Target | Concentration | Metal | Clone | cat #
ICOS | 1:200 | 111Cd | C398.4A | BioLegend, cat # 313502
Cd15s | 1:200 | 113Cd | CSLEX1 | BD Biosciences, cat # 551344
CD39 | 1:200 | 116Cd | A1 | BioLegend, cat #328221
CTLA-4 | 1:100 | 142Nd | 14D3 | Invitrogen, cat #14-1529-82
TIGIT | 1:200 | 159Tb | VSTM3 | BioLegend, cat # 372702
CCR10 | 1:100 | 169Tm | 6588-5 | BioLegend, cat # 341502

Validation

Maxpar Direct Immune Profiling assay (Standard BioTools, cat # 201325), validated 30-marker assay; reactivity: human, validated for human whole blood and PBMC samples, proven performance and reproducibility: DOI: 10.1126/sciimmunol.abf3733
Target | Concentration | Metal | Clone | cat # |Validation
LAG-3 | 1:100 | 165Ho | 11C3C65 | St. BioTools, cat # 3165037B | reactivity: human; quality-control tested for CyTOF application, DOI: 10.1016/j.jim.2010.07.002, DOI: 10.3389/fimmu.2023.1145814
CD69 | 1:200 | 162Dy | FN50 | St. BioTools, cat # 3162001B | reactivity: human Cynomolgus Monkey, Rhesus; quality-control tested for CyTOF application, DOI: 10.1016/j.celrep.2019.01.085
PD-1 | 1:200 | 175Lu | EH12.2H7 | St. BioTools, cat # 3175008B| reactivity: human Cynomolgus Monkey, Rhesus; quality-control tested for CyTOF application, doi: 10.1016/j.xpro.2022.101362
ICOS | 1:200 | 111Cd | C398.4A | BioLegend, cat # 313502 | verified reactivity: human, mouse, rat; tested for flow cytometry, verified for flow cytometry: DOI: 10.4049/jimmunol.171.2.783, DOI: 10.1038/ni.3793
Cd15s | 1:200 | 113Cd | CSLEX1 | BD Biosciences, cat # 551344 | verified reactivity: human; routinely tested for flow cytometry, DOI: 10.1371/journal.pone.0070139
CD39 | 1:200 | 116Cd | A1 | BioLegend, cat #328221 | verified reactivity: Human, Cynomolgus, Rhesus; tested for flow cytometry, verified for CyTOF, Immunohistochemistry-paraffin: DOI: 10.1016/j.xpro.2022.101643, DOI: 10.1016/j.cell.2020.09.034
CTLA-4 | 1:100 | 142Nd | 14D3 | Invitrogen, cat #14-1529-82 | verified reactivity: Human, mouse; tested for flow cytometry, verified for flow cytometry, western blot: DOI: 10.1038/s41467-021-21078-7, DOI: 10.1371/journal.pone.0112509
TIGIT | 1:200 | 159Tb | VSTM3 | BioLegend, cat # 372702 | verified reactivity: human; tested for flow cytometry and blocking: DOI: 10.1038/s41388-018-0288-y
CCR10 | 1:100 | 169Tm | 6588-5 | BioLegend, cat # 341502 | verified reactivity: human; tested for flow cytometry, verified for CITE-seq experiment: DOI: 10.1016/j.medj.2021.01.006.

Eukaryotic cell lines

Policy information about cell lines and Sex and Gender in Research

Cell line source(s)

State the source of each cell line used and the sex of all primary cell lines and cells derived from human participants or vertebrate models.

Authentication

Describe the authentication procedures for each cell line used OR declare that none of the cell lines used were authenticated.

Mycoplasma contamination

Confirm that all cell lines tested negative for mycoplasma contamination OR describe the results of the testing for mycoplasma contamination OR declare that the cell lines were not tested for mycoplasma contamination.

Commonly misidentified lines
(See ICLAC register)

Name any commonly misidentified cell lines used in the study and provide a rationale for their use.

Palaeontology and Archaeology

Specimen provenance

Provide provenance information for specimens and describe permits that were obtained for the work (including the name of the issuing authority, the date of issue, and any identifying information). Permits should encompass collection and, where applicable, export.

Specimen deposition

Indicate where the specimens have been deposited to permit free access by other researchers.

Dating methods

If new dates are provided, describe how they were obtained (e.g. collection, storage, sample pretreatment and measurement), where they were obtained (i.e. lab name), the calibration program and the protocol for quality assurance OR state that no new dates are provided.

☐ Tick this box to confirm that the raw and calibrated dates are available in the paper or in Supplementary Information.

Ethics oversight

Identify the organization(s) that approved or provided guidance on the study protocol, OR state that no ethical approval or guidance

Ethics oversight | *was required and explain why not.*

Note that full information on the approval of the study protocol must also be provided in the manuscript.

Animals and other research organisms

Policy information about studies involving animals; ARRIVE guidelines recommended for reporting animal research, and Sex and Gender in Research

Laboratory animals | *For laboratory animals, report species, strain and age OR state that the study did not involve laboratory animals.*

Wild animals | *Provide details on animals observed in or captured in the field; report species and age where possible. Describe how animals were caught and transported and what happened to captive animals after the study (if killed, explain why and describe method; if released, say where and when) OR state that the study did not involve wild animals.*

Reporting on sex | *Indicate if findings apply to only one sex; describe whether sex was considered in study design, methods used for assigning sex. Provide data disaggregated for sex where this information has been collected in the source data as appropriate; provide overall numbers in this Reporting Summary. Please state if this information has not been collected. Report sex-based analyses where performed, justify reasons for lack of sex-based analysis.*

Field-collected samples | *For laboratory work with field-collected samples, describe all relevant parameters such as housing, maintenance, temperature, photoperiod and end-of-experiment protocol OR state that the study did not involve samples collected from the field.*

Ethics oversight | *Identify the organization(s) that approved or provided guidance on the study protocol, OR state that no ethical approval or guidance was required and explain why not.*

Note that full information on the approval of the study protocol must also be provided in the manuscript.

Clinical data

Policy information about clinical studies

All manuscripts should comply with the ICMJE guidelines for publication of clinical research and a completed CONSORT checklist must be included with all submissions.

Clinical trial registration | *Provide the trial registration number from ClinicalTrials.gov or an equivalent agency.*

Study protocol | *Note where the full trial protocol can be accessed OR if not available, explain why.*

Data collection | *Describe the settings and locales of data collection, noting the time periods of recruitment and data collection.*

Outcomes | *Describe how you pre-defined primary and secondary outcome measures and how you assessed these measures.*

Dual use research of concern

Policy information about dual use research of concern

Hazards

Could the accidental, deliberate or reckless misuse of agents or technologies generated in the work, or the application of information presented in the manuscript, pose a threat to:

No	Yes	
☐	☐	Public health
☐	☐	National security
☐	☐	Crops and/or livestock
☐	☐	Ecosystems
☐	☐	Any other significant area

Experiments of concern

Does the work involve any of these experiments of concern:

No	Yes	
☐	☐	Demonstrate how to render a vaccine ineffective
☐	☐	Confer resistance to therapeutically useful antibiotics or antiviral agents
☐	☐	Enhance the virulence of a pathogen or render a nonpathogen virulent
☐	☐	Increase transmissibility of a pathogen
☐	☐	Alter the host range of a pathogen
☐	☐	Enable evasion of diagnostic/detection modalities
☐	☐	Enable the weaponization of a biological agent or toxin
☐	☐	Any other potentially harmful combination of experiments and agents

Plants

Seed stocks

Report on the source of all seed stocks or other plant material used. If applicable, state the seed stock centre and catalogue number. If plant specimens were collected from the field, describe the collection location, date and sampling procedures.

Novel plant genotypes

Describe the methods by which all novel plant genotypes were produced. This includes those generated by transgenic approaches, gene editing, chemical/radiation-based mutagenesis and hybridization. For transgenic lines, describe the transformation method, the number of independent lines analyzed and the generation upon which experiments were performed. For gene-edited lines, describe the editor used, the endogenous sequence targeted for editing, the targeting guide RNA sequence (if applicable) and how the editor was applied.

Authentication

Describe any authentication procedures for each seed stock used or novel genotype generated. Describe any experiments used to assess the effect of a mutation and, where applicable, how potential secondary effects (e.g. second site T-DNA insertions, mosiacism, off-target gene editing) were examined.

ChIP-seq

Data deposition

☐ Confirm that both raw and final processed data have been deposited in a public database such as GEO.

☐ Confirm that you have deposited or provided access to graph files (e.g. BED files) for the called peaks.

Data access links
May remain private before publication.

For "Initial submission" or "Revised version" documents, provide reviewer access links. For your "Final submission" document, provide a link to the deposited data.

Files in database submission

Provide a list of all files available in the database submission.

Genome browser session
(e.g. UCSC)

Provide a link to an anonymized genome browser session for "Initial submission" and "Revised version" documents only, to enable peer review. Write "no longer applicable" for "Final submission" documents.

Methodology

Replicates

Describe the experimental replicates, specifying number, type and replicate agreement.

Sequencing depth

Describe the sequencing depth for each experiment, providing the total number of reads, uniquely mapped reads, length of reads and whether they were paired- or single-end.

Antibodies

Describe the antibodies used for the ChIP-seq experiments; as applicable, provide supplier name, catalog number, clone name, and lot number.

Peak calling parameters

Specify the command line program and parameters used for read mapping and peak calling, including the ChIP, control and index files used.

Data quality

Describe the methods used to ensure data quality in full detail, including how many peaks are at FDR 5% and above 5-fold enrichment.

Software

Describe the software used to collect and analyze the ChIP-seq data. For custom code that has been deposited into a community repository, provide accession details.

Flow Cytometry

Plots

Confirm that:

☒ The axis labels state the marker and fluorochrome used (e.g. CD4-FITC).

☒ The axis scales are clearly visible. Include numbers along axes only for bottom left plot of group (a 'group' is an analysis of identical markers).

☒ All plots are contour plots with outliers or pseudocolor plots.

☒ A numerical value for number of cells or percentage (with statistics) is provided.

Methodology

Sample preparation	Upon thawing, PBMCs were washed with FACS buffer (PBS, 2%FBS, 0,1%NaN3 sodium azide). Further, for CD161 detection in NK cells, PBMCs were incubated with the following antibody cocktail for 30 minutes at +4C in the dark (anti-human CD45 FITC (1:100, clone HI30, BioLegend, cat # 304006), anti-human CD19 PE (1:100, clone HIB19, eBioscience, cat # 12-0199-42), anti-human CD3 PerCP-Cy5.5 (1:100, clone UCHT1, BD Biosciences, cat # 560835), anti-human CD14 PE-Cy7 (1:100, clone 63D3, BioLegend, cat # 367112), anti-human CD56 BV421 (1:100, clone NCAM16.2, BD Biosciences, cat # 562751), anti-human CD161 APC (1:100, clone HP-3G10, BioLegend, cat # 339912). For CD39 detection in memory Treg cells, CD4+ T cells were isolated from PBMCs using human CD4 T cells kit (Dynabeads, Thermo Fisher). Next, isolated CD4+ T cells were incubated with the following antibody cocktail for 30 minutes at +4C in the dark (Anti-human CD3 PerCP-Cy5.5 (1:100, clone UCHT1, BD Biosciences, cat # 560835), anti-human CD4 BV421 (1:100, clone RPA-T4, BD Biosciences, cat # 562424), anti-human CD25 BB515 (1:20, clone BC96, BD Biosciences, cat # 567318), anti-human CD127 PE-Cy7 (1:100, clone A019D5, BioLegend, cat # 351320), anti-human CD45RA BV786 (1:100, clone HI100, BioLegend, cat # 304140), anti-human CD45RO PE (1:100, clone UCHL1, BD Biosciences, cat # 555493), anti-human CD39 APC (1:100, clone A1, BioLegend, cat # 328210). For both applications, staining with Fixable Viability Dye eFluor 780 (cat. 65-0865-14, eBioscience, Thermo Fisher Scientific, USA) was done after surface staining with antibodies. Further, cells were washed 2 times with FACS buffer. Data were acquired on the BD LSRFortessa™ Cell Analyzer flow cytometer (BD Biosciences, USA) with BD FACSDiva™ software v. 9.
Instrument	Flow cytometry was performed on LSRFortessa flow cytometer (BD Biosciences)
Software	BD FACSDiva™ Software v. 9 and FCS Express 7 Flow version 7.16.0046
Cell population abundance	The cell population abundance varied between the samples.
Gating strategy	For CD161 expression in NK cells the gating was as follows: FSC/SSC to exclude outliers; e780 Viability Dye– and CD45+; further, NK cell population was defined as CD3–CD14–CD19–CD56+<br>For CD39 expression in Treg cells the gating was as follows: FSC/SSC to exclude outliers; e780 Viability Dye– CD3+ CD4+; further, Treg cell population was defined as CD25+CD127low; CD45RO and CD45RA were used to define memory and naive Tregs. Gating strategies are shown in Supplementary Figures 3, 4.

☒ Tick this box to confirm that a figure exemplifying the gating strategy is provided in the Supplementary Information.

Magnetic resonance imaging

Experimental design

Design type	*Indicate task or resting state; event-related or block design.*
Design specifications	*Specify the number of blocks, trials or experimental units per session and/or subject, and specify the length of each trial or block (if trials are blocked) and interval between trials.*
Behavioral performance measures	*State number and/or type of variables recorded (e.g. correct button press, response time) and what statistics were used to establish that the subjects were performing the task as expected (e.g. mean, range, and/or standard deviation across subjects).*

Acquisition

Imaging type(s)	*Specify: functional, structural, diffusion, perfusion.*
Field strength	*Specify in Tesla*
Sequence & imaging parameters	*Specify the pulse sequence type (gradient echo, spin echo, etc.), imaging type (EPI, spiral, etc.), field of view, matrix size, slice thickness, orientation and TE/TR/flip angle.*
Area of acquisition	*State whether a whole brain scan was used OR define the area of acquisition, describing how the region was determined.*

Diffusion MRI ☐ Used ☐ Not used

Preprocessing

Preprocessing software	*Provide detail on software version and revision number and on specific parameters (model/functions, brain extraction, segmentation, smoothing kernel size, etc.).*
Normalization	*If data were normalized/standardized, describe the approach(es): specify linear or non-linear and define image types used for transformation OR indicate that data were not normalized and explain rationale for lack of normalization.*
Normalization template	*Describe the template used for normalization/transformation, specifying subject space or group standardized space (e.g. original Talairach, MNI305, ICBM152) OR indicate that the data were not normalized.*
Noise and artifact removal	*Describe your procedure(s) for artifact and structured noise removal, specifying motion parameters, tissue signals and physiological signals (heart rate, respiration).*
Volume censoring	*Define your software and/or method and criteria for volume censoring, and state the extent of such censoring.*

Statistical modeling & inference

Model type and settings	*Specify type (mass univariate, multivariate, RSA, predictive, etc.) and describe essential details of the model at the first and second levels (e.g. fixed, random or mixed effects; drift or auto-correlation).*
Effect(s) tested	*Define precise effect in terms of the task or stimulus conditions instead of psychological concepts and indicate whether ANOVA or factorial designs were used.*

Specify type of analysis: ☐ Whole brain ☐ ROI-based ☐ Both

Statistic type for inference<br>(See Eklund et al. 2016)	*Specify voxel-wise or cluster-wise and report all relevant parameters for cluster-wise methods.*
Correction	*Describe the type of correction and how it is obtained for multiple comparisons (e.g. FWE, FDR, permutation or Monte Carlo).*

Models & analysis

n/a	Involved in the study
☐	☐ Functional and/or effective connectivity
☐	☐ Graph analysis
☐	☐ Multivariate modeling or predictive analysis

Functional and/or effective connectivity	*Report the measures of dependence used and the model details (e.g. Pearson correlation, partial correlation, mutual information).*
Graph analysis	*Report the dependent variable and connectivity measure, specifying weighted graph or binarized graph, subject- or group-level, and the global and/or node summaries used (e.g. clustering coefficient, efficiency, etc.).*
Multivariate modeling and predictive analysis	*Specify independent variables, features extraction and dimension reduction, model, training and evaluation metrics.*
